# A Rare Case of Paraneoplastic Aortitis Associated with Chronic Myelomonocytic Leukemia

**DOI:** 10.1155/2017/3091973

**Published:** 2017-05-25

**Authors:** Sylwia Sasinowska, Pamela Traisak, Michael McCormack, Hala Eid

**Affiliations:** ^1^Division of Rheumatology, Cooper University Hospital, Camden, NJ, USA; ^2^Division of Hematology-Oncology, MD Anderson Cancer Center, Cooper University Hospital, Camden, NJ, USA

## Abstract

Aortitis is a broad term describing inflammation of the aorta. The most common causes of aortitis are the large-vessel vasculitides giant cell arteritis and Takayasu arteritis. Other etiologies include aortitis associated with other autoimmune disorders, infectious causes, and paraneoplastic and idiopathic cases. We describe a rare case of a large-vessel arteritis occurring in association with chronic myelomonocytic leukemia (CMML). A 68-year-old female with recent diagnosis of CMML presented to our office for evaluation of abnormal chest computed tomography (CT) that showed inflammation surrounding the entirety of thoracic and abdominal aorta, consistent with aortitis. In the absence of other evident causes of large-vessel vasculitis, we attributed this finding to a paraneoplastic autoimmune phenomenon and started treatment with systemic glucocorticoids. This rare case emphasizes the need to recognize autoimmune complications in CMML and treat the inflammation along with the primary malignancy promptly.

## 1. Introduction

Aortitis is a large-vessel vasculitis involving inflammation of the aortic wall. The most common causes of aortitis are giant cell arteritis and Takayasu arteritis, although it has been linked with several other conditions [1]. There are very few case reports in the literature of large-vessel arteritis associated with myelodysplastic and myeloproliferative (MDS/MPD) syndromes, particularly with chronic myelomonocytic leukemia (CMML) [2–4]. The vasculitides occurring with MDS usually involve small cutaneous vessels, less commonly medium-sized arteries, and infrequently large arteries such as the aorta [5]. We report a rare case of large-vessel vasculitis occurring in association with CMML.

## 2. Case Presentation

A 68-year-old Caucasian woman recently diagnosed with CMML presented to our rheumatology office for evaluation of an abnormal chest computed tomography (CT) showing inflammation surrounding the entire thoracic and abdominal aorta. She was experiencing shortness of breath, fatigue, fevers, and night sweats for about four months previously. She additionally had an unintentional weight loss of 35 pounds over the past 2 years which was initially attributed to Nissen fundoplication procedure for gastroesophageal reflux. She denied any headaches, vision changes, scalp tenderness, jaw pain, dysphagia, joint pain, joint swelling, muscle pain, or photosensitivity.

For the past eight months, she had been undergoing hematological workup of abnormal blood counts. Her white blood cell (WBC) counts were elevated with monocytosis and varying degrees of neutrophilia over the past 3 years. She had thrombocytosis. Her initial evaluation was negative for BCR-ABL PCR testing, Janus kinase-2, calreticulin, and MPL mutations. There was no hepatosplenomegaly on imaging. After these tests returned negative, she was planned for a bone marrow biopsy for further evaluation of monocytosis but opted to wait as she was asymptomatic. However, she presented back with a nontender, erythematous lesion on her left breast. About two weeks later, similar scattered pink small dermal papulonodules were present on the posterior neck, left shoulder, abdomen, and left thigh. Left breast punch biopsy described a dense dermal infiltrate with small lymphocytes with cytologic atypia. There was no significant epidermal involvement. The lymphocytes were positive for CD4 and CD43, myeloperoxidase, and lysozyme. The overall morphologic and immunohistochemical findings were those of a malignant neoplasm. Although this was originally thought to be a possible T cell lymphoma, case was discussed at the hematopathology conference and the overall presentation correlated with more systemic process, such as CMML.

**Figure 1:**
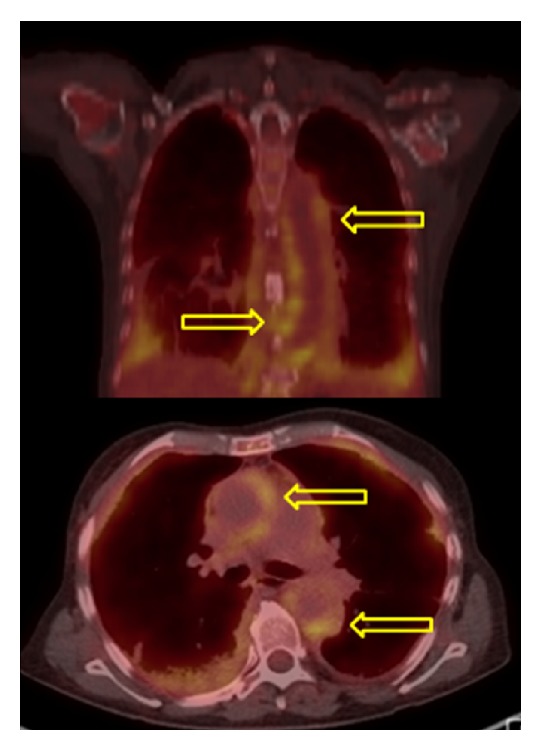
PET scan showing nodular FDG uptake in the wall of the thoracic and upper abdominal aorta and in the right atrium, suspicious for large cell arteritis.

**Figure 2:**
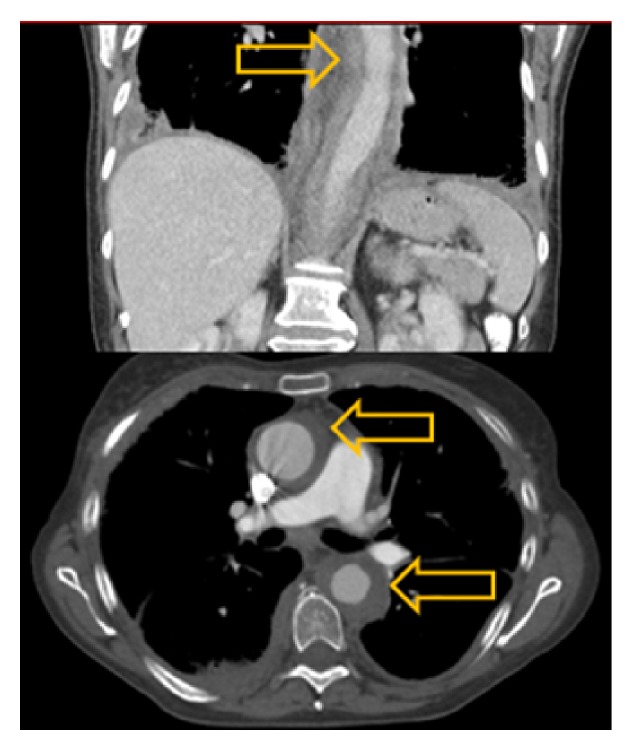
Chest CT with IV contrast showing soft tissue density surrounding the entirety of the thoracic and abdominal aorta (arrows), consistent with large-vessel vasculitis.

Subsequent bone marrow biopsy and aspiration showed pieces of hypercellular bone marrow with overall cellularity of 50%, trilineage hematopoiesis, hypogranular myeloid cells, and increase of monocytoid cells with somewhat immature appearing features. Increased monocytosis (20%) with oval-shaped, slightly irregular nuclei was present. There was no evidence of fibrosis on reticulin staining. Adequate megakaryopoiesis and erythropoiesis without dysplastic changes was noted. The myeloid-to-erythroid ratio was mildly increased. Cytogenetic evaluation exhibited a normal karyotype (46, XX). Flow cytometry revealed CD14+/CD16- monocytes that are characteristic of CMML [6]. CMML diagnosis was made based on the above findings, persistent peripheral blood monocytosis ≥ 1 × 10^9^/L, with monocytes accounting for ≥10% of the WBC count, no evidence of PDGFRA/PDGFRB, <20% blasts in the blood and bone marrow, not meeting WHO criteria for BCR-ABL1 positive chronic myeloid leukemia, primary myelofibrosis, polycythemia vera, or essential thrombocytosis and all other causes of monocytosis excluded [7]. For staging, she underwent positron emission tomography (PET) and computed tomography (CT) scans and both imaging studies showed soft tissue density surrounding the entirety of the thoracic and abdominal aorta, consistent with large-vessel vasculitis (see Figures 1 and 2).

Laboratory studies were positive for antinuclear antibody (ANA) with a titer of 1 : 640 in a nucleolar and speckled pattern as well as elevated RNP antibody (ab) at 1.1. Her inflammatory markers were elevated: erythrocyte sedimentation rate (ESR) 36 mm/hr and C-reactive protein (CRP) 35.3 mg/L. The negative serologies included an anti-neutrophil cytoplasmic ab, anti-proteinase 3 ab, anti-myeloperoxidase ab, SS-A/SS-B ab, anti-Smith ab, anti-dsDNA ab, anti-smooth muscle ab, rheumatoid factor, anti-cyclic citrullinated peptide ab, and hepatitis panel.

For symptomatic aortitis, we started treatment with prednisone 1 mg/kg/day. Within a week, patient had significant improvement. Her shortness of breath subsided and she felt strong and energetic. Objectively, while on steroid therapy, her WBC count initially dropped. Since the aortitis related symptoms were improving, the hematology team planned to start her on treatment with a hypomethylating agent—azacitidine.

Steroid therapy was slowly tapered given her good response to initial high-dose steroids. However, two months later, she developed steroid-induced myopathy and prednisone was tapered more quickly. Within two weeks, she became more dyspneic and hypoxic. Her respiratory status deteriorated quickly, requiring intubation and mechanical ventilation. She was treated with broad-spectrum antibiotics for possible infectious etiology. CT chest demonstrated stable aortitis but increased diffuse ground-glass opacity throughout both lungs and bilateral pulmonary consolidation. These findings were attributed to paraneoplastic infiltrates from leukemia and less likely to be infectious. Cultures from bronchoalveolar lavage and* Pneumocystis jirovecii* testing were negative. Coincidentally, prior to this admission, she had a stable WBC count of 18,600/*μ*L that now has progressed to WBC count of 77,000/*μ*L, despite negative infectious workup, aortitis being asymptomatic and stable on CT imaging study.

Given her clinical deterioration from leukemia, in her final days she received her first treatment of CMML with hydroxyurea, then underwent leukapheresis, and received one dose of chemotherapy with decitabine one day prior to expiration. Unfortunately, she continued to deteriorate and was placed on hospice. Her family refused an autopsy.

## 3. Discussion

In the present case, our patient developed aortitis associated with CMML. CMML is a clonal disorder of a bone marrow stem cell with monocytosis as a major defining feature. Initially, it was classified as a purely myelodysplastic disorder. However, clinicians now recognize that CMML displays both dysplastic and proliferative features [8]. Because of these dual characteristics, the WHO classifies CMML as a myelodysplastic/myeloproliferative disease (MDS/MPD) [7, 9]. Approximately 10–20% of patients with MDS present with systemic inflammatory and autoimmune diseases (SIADs) [10, 11]. Vasculitis is an unusual but documented paraneoplastic complication of MDS and MPD disorders—affecting mainly small and medium vessels and, very rarely, large vessels such as the aorta. Vasculitides occurring with MDS/MPD predominantly present with cutaneous lesions, arthralgias, and neuropathy [5]. Leukocytoclastic vasculitis and polyarteritis nodosa are most frequently associated with MDS [12]. Our literature search found only three case reports describing aortitis complicating specifically CMML [2–4]. Immunological abnormalities have been reported in MDS/MPD syndromes and are characterized both by humoral and by cellular abnormalities [13]. Although the exact underlying mechanisms remain unclear, it has been hypothesized that deficiencies in lymphocyte function, defective natural killer cells, faulty phagocytic cell function, and dysregulated antigenic presentation with ongoing immune stimulation may play a role [4].

In our patient, acute systemic inflammatory symptoms were alleviated almost immediately with steroid therapy. However, patient's CMML progressed very quickly and she died from leukemia-related complications within three months of her aortitis diagnosis. In a French multicenter retrospective study, the diagnosis of SIADs associated with MDS preceded MDS in 37% of cases, was concomitant in 31% of cases, and occurred after MDS diagnosis in 32% of cases, with a mean of 8.6 months between the diagnoses of the two disorders [14].

The initial treatment of vasculitis consists of corticosteroids and specific treatment of MDS/MPD syndromes. One study on 26 patients with CMML complicated by SIADs revealed an early response to steroids alone in 87% of cases. However, despite this initial response, 40% of patients needed a second-line treatment for relapse or steroid dependence [8]. Another larger study had similar results for MDS-associated SIADs, in which 80% of patients responded to corticosteroid alone and 50% of patients required a second-line treatment for similar reasons as above [14]. Treatment with hypomethylating agents, azacitidine and decitabine, showed added response in majority of cases of CMML and other MDS patients, with significantly decreased amounts and steroid dependence rates [8, 10].

## 4. Conclusion

Large-vessel vasculitis is a rare but significant complication of CMML that contributes to the morbidity and mortality of the disease. As with other paraneoplastic autoimmune diseases, vasculitis initially responds to corticosteroid therapy but eventually requires specific treatment of the underlying malignancy.
